# 1251. Comparative Evaluation of Phenotypic Synergy Tests vs. Resist-4 O.K.N.V^®^ and NG Test Carba 5^®^ Lateral Flow Immunoassays for the Detection and Differentiation of Carbapenemases in *Enterobacterales* and *Pseudomonas aeruginosa*

**DOI:** 10.1093/ofid/ofab466.1443

**Published:** 2021-12-04

**Authors:** Diego Josa, Rafael Leal, Julieth Rojas, Isabel Torres, Fabian Cortes, German Esparza, Luis Reyes

**Affiliations:** 1 Fundación Clínica Shaio, Bogota, Cundinamarca, Colombia; 2 Clínica Shaio, Bogota, Distrito Capital de Bogota, Colombia; 3 Proasecal SAS, Bogota, Distrito Capital de Bogota, Colombia; 4 Universidad de la Sabana, Bogota, Distrito Capital de Bogota, Colombia

## Abstract

**Background:**

The spread of carbapenem resistant *Pseudomonas aeruginosa* and carbapenemase-producing *Enterobacterales* (CPE) has had a great impact on morbidity and mortality. COVID-19 pandemic has favoured the selection of these microorganisms because of the excessive and prolonged use of broad-spectrum antibiotics and the outbreaks related to patient transfer between hospitals and inadequate use of personal protective equipment. Therefore, detection is considered essential for their control. Our aim was to compare conventional phenotypic synergy tests and two lateral flow immunoassays for detecting carbapenemases in *Enterobacterales* and *P. aeruginosa.*

**Methods:**

We analysed 100 carbapenem-resistant Gram-negative bacilli isolates, 80 *Enterobacterales* and 20 *Pseudomonas aeruginosa*, (86 isolates producing KPC, NDM, OXA-48, IMP and VIM carbapenemases and 14 non-carbapenemase-producing isolates). We performed a modified Hodge test, boronic acid and ethylenediaminetetraacetic acid (EDTA) synergy tests, and two lateral flow immunoassays: RESIST-4 O.K.N.V (Coris Bioconcept®) and NG Test Carba 5® (NG Biotech®).

**Results:**

In total, 76 KPC, 7 VIM, 1 NDM, 1 OXA-48 and 1 isolate coproducing KPC + NDM enzymes were included. The concordance of different methods estimated by Kappa index was 0.432 (Standard error: 0.117), thus showing a high variability with the synergy tests with boronic acid and EDTA and reporting 16 false negatives that were detected by the two immunochromatographic methods. Co-production was only detected using immunoassays.

Figure 1. Errors in synergy tests.

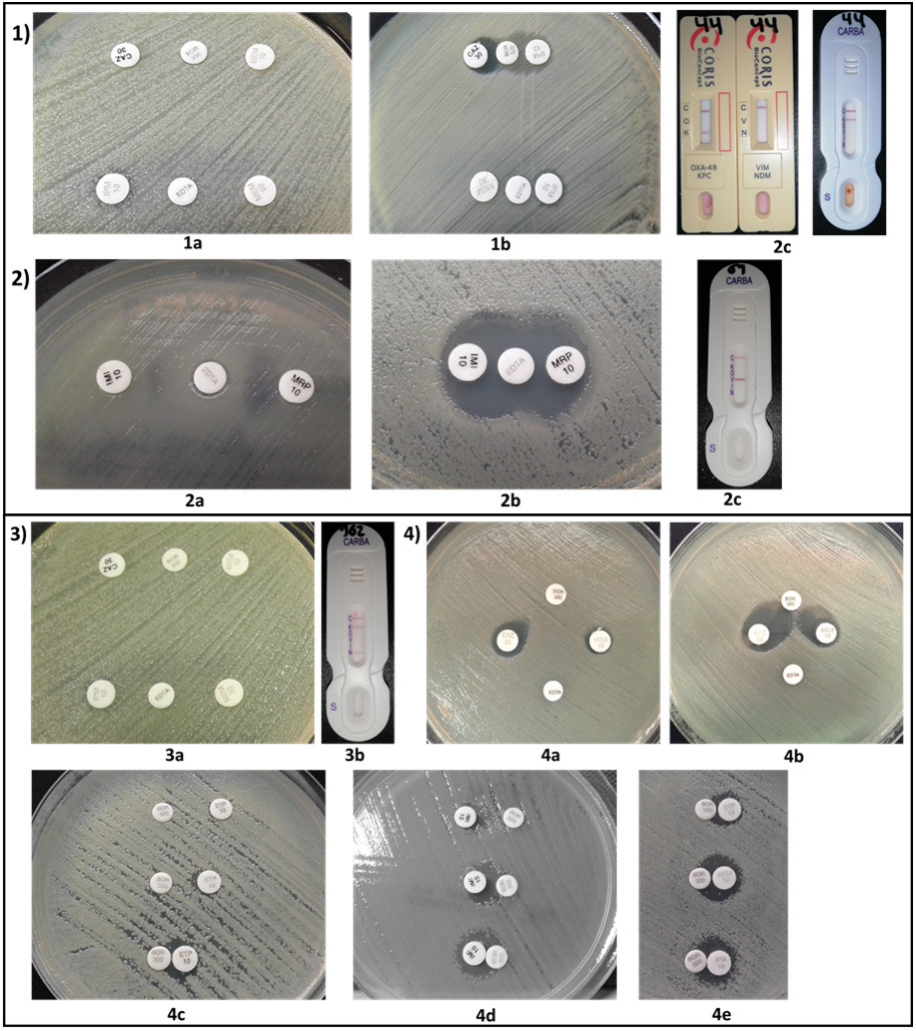

1) KPC-producing K. pneumoniae not detected with boronic acid and EDTA (1a). Positive result adjusting distance to 5 mm (1b) and positive result by NG Test Carba 5® and RESIST-4 O.K.N.V® immunoassays (1c). 2) VIM -producing P. aeruginosa with inconclusive EDTA result (2a), Positive result adjusting distance to 5 mm (2b), confirmation of VIM by immunoassay (2c). 3) K. pneumoniae with KPC and NDM co-production without synergistic effect with boronic acid or EDTA (3a), positivity for both enzymes by immunoassay (3b). 4) Impact of distance between disks: False negative for serine-carbapenemases with 15 mm (4a) positive result at 10 mm (4b). Differences in boronic acid tests for serin-carbapenemases at 10 mm, 5 mm and 0 mm distance (4c, 4d). Differences in synergy between imipenem, meropenem and ertapenem discs at 0 mm distance (4e).

Table 2. Results of synergy methods and immunoassay tests for Enterobacterales and Pseudomonas aeruginosa

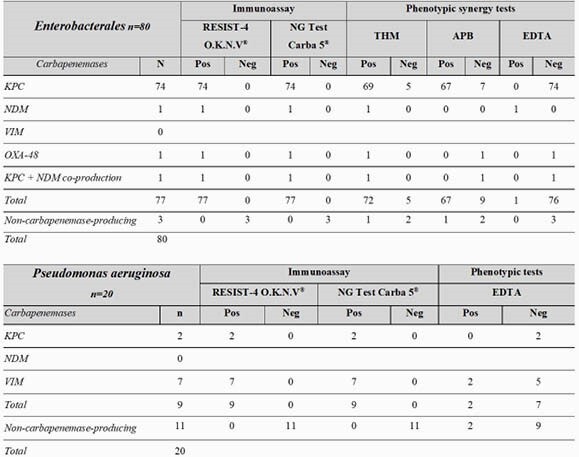

**MHT:**

Modified Hodge Test, APB: boronic acid synergy; EDTA: EDTA synergy; Pos: positive; Neg: negative. KPC (Klebsiella pneumoniae Carbapenemase), VIM (Verona integron-mediated metallo- β-lactamase), NDM (New Delhi metallo-β-lactamase), OXA (oxacillinase-48-like carbapenemase (OXA-48))

**Conclusion:**

Conventional phenotypic synergy tests with boronic acid and EDTA used for detecting carbapenemases are suboptimal and their routine use should be reconsidered. They depend on the degree of enzyme expression and the distance between disks. Lateral flow immunoassay tests are a rapid and cost-effective tool to detect and differentiate carbapenemases, improving clinical outcomes through targeted therapy and promoting infection prevention measures.

**Disclosures:**

**Diego Josa, Msc**, **ALIFAX** (Speaker’s Bureau) **German Esparza, n/a**, **Biomerieux** (Consultant)**Pfizer** (Speaker’s Bureau) **Luis Reyes, n/a**, **MSD** (Speaker’s Bureau)

